# Comparative Transcriptomic Analyses of Nitrate-Response in Rice Genotypes With Contrasting Nitrogen Use Efficiency Reveals Common and Genotype-Specific Processes, Molecular Targets and Nitrogen Use Efficiency-Candidates

**DOI:** 10.3389/fpls.2022.881204

**Published:** 2022-06-14

**Authors:** Narendra Sharma, Supriya Kumari, Dinesh Kumar Jaiswal, Nandula Raghuram

**Affiliations:** University School of Biotechnology, Guru Gobind Singh Indraprastha University, New Delhi, India

**Keywords:** networks, nitrate, nitrogen use efficiency, QTLs, rice, transcriptome

## Abstract

The genetic basis for nitrogen (N)-response and N use efficiency (NUE) must be found in N-responsive gene expression or protein regulation. Our transcriptomic analysis of nitrate response in two contrasting rice genotypes of *Oryza sativa* ssp. *Indica* (Nidhi with low NUE and Panvel1 with high NUE) revealed the processes/functions underlying differential N-response/NUE. The microarray analysis of low nitrate response (1.5 mM) relative to normal nitrate control (15 mM) used potted 21-days old whole plants. It revealed 1,327 differentially expressed genes (DEGs) exclusive to Nidhi and 666 exclusive to Panvel1, apart from 70 common DEGs, of which 10 were either oppositely expressed or regulated to different extents. Gene ontology analyses revealed that photosynthetic processes were among the very few processes common to both the genotypes in low N response. Those unique to Nidhi include cell division, nitrogen utilization, cytoskeleton, etc. in low N-response, whereas those unique to Panvel1 include signal transduction, protein import into the nucleus, and mitochondria. This trend of a few common but mostly unique categories was also true for transporters, transcription factors, microRNAs, and post-translational modifications, indicating their differential involvement in Nidhi and Panvel1. Protein-protein interaction networks constructed using DEG-associated experimentally validated interactors revealed subnetworks involved in cytoskeleton organization, cell wall, etc. in Nidhi, whereas in Panvel1, it was chloroplast development. NUE genes were identified by selecting yield-related genes from N-responsive DEGs and their co-localization on NUE-QTLs revealed the differential distribution of NUE-genes between genotypes but on the same chromosomes 1 and 3. Such hotspots are important for NUE breeders.

## Introduction

Reactive nitrogen (N) impacts all 17 sustainable development goals including food security. It is quantitatively the most important nutritional requirement for plant growth and agricultural productivity and is therefore supplied in various organic and inorganic forms including urea, ammonium salts, and nitrates. However, poor nitrogen use efficiency (NUE) in agriculture is one of the major reasons for anthropogenic nitrogen pollution that affects soil, air, water, health, biodiversity, and climate change (Sutton et al., 2019). It has already crossed our planetary boundaries, in addition to the loss of fertilizers worth billions of dollars (Sutton et al., 2019). The advocacy of the International Nitrogen Initiative and others over the last two decades led to the UNEP resolution on Sustainable Nitrogen Management in 2019 (Raghuram et al., 2021). Improving agricultural NUE is critical to meet the emerging calls to halve the nitrogen waste (Sutton et al., 2021), especially in agrarian countries.

Plant biology has a central role in understanding and improving crop NUE (Raghuram and Sharma, 2019; Udvardi et al., 2021; Madan et al., 2022). This has to begin with cereals that dominate global crop production and fertilizer demand, of which rice is predominant, due to its lowest NUE (Norton et al., 2015). It is the third most-produced and second most consumed crop in the world, apart from being a post-genomic model crop. This is evidenced by genome sequences of 3000 rice genotypes (Li et al., 2014) and growing functional genomics of N (Li et al., 2018; Pathak et al., 2020; Mandal et al., 2022). Further, the recent NUE phenotype (Sharma et al., 2018, 2021; Pathak et al., 2021) growing quantitative trait loci (QTL) and genotyping (Kumari et al., 2021) make rice an ideal target crop for NUE improvement. A recent simulation showed that $743 million per year could be saved by a 20% increase in rice NUE (Langholtz et al., 2021), while the global expenditure in that direction is not even a tiny fraction of it. Therefore, improving rice NUE is a highly desirable economic and environmental goal.

Nitrogen use efficiency can be understood in terms of uptake/utilization or remobilization efficiencies but is agronomically best expressed as yield or harvested N per unit N input (Raghuram and Sharma, 2019). Among the various N-fertilizers used as inputs, urea is the most predominant form of N-fertilizer used in the rice-growing and developing countries, whereas nitrates and ammonium salts are predominant in cropping in the developed world. However, soil microbial conversions ensure that nitrate is the predominant form of N available to all crops including rice, regardless of the form of N-supply (Coskun et al., 2017). This is one of the reasons why nitrate-transcriptomes are predominant even in the rice functional genomics literature (listed in Kumari et al., 2021). They are also available for subspecies Indica (Pathak et al., 2020) and Japonica (Mandal et al., 2022).

Nitrate uptake is mediated by nitrate transporters followed by intracellular conversion into ammonium ions by the sequential action of nitrate reductase (*NR*) and nitrite reductase (*NiR*) and assimilated into amino acids by the glutamine synthetase and glutamate synthase (*GS-GOGAT*) cycle (Raghuram and Sharma, 2019). All other metabolites containing N are generated by transamination of amino acids, which also provide the main organic N-pool for translocation and secondary remobilization during senescence, which is particularly important in cereals (Snyder and Tegeder, 2021). All these processes have been targeted for understanding and improving NUE with varying results (Mandal et al., 2018; Raghuram and Sharma, 2019; Sinha et al., 2016, 2020; Madan et al., 2022).

In rice, several genes such as *OsGRF4, OsDof1, NADH-GOGAT, OsNPF6.1, OsNRT2.3b, OsNRT2.1, OsPTR9, OsNPF8.20, OsNRT1.1A, OsFBP1* have been reported to increase NUE (Kumari et al., 2021 and references cited therein) including alanine aminotransferase (Good et al., 2007), *OsPTR9* (Fang et al., 2013), and *DEP1* (Sun et al., 2014). In addition to these genes, some interesting QTLs have been identified as linked to NUE (Sinha et al., 2018; Waqas et al., 2018; Kumari et al., 2021). Transcriptomic studies in rice revealed thousands of nitrate-responsive genes totaling 23,626 numbers (Mandal et al., 2022) but they were all limited to single genotypes. This limits the utility of functional genomic studies in the genetic dissection of NUE. The only studies that compared the transcriptomes of two genotypes for NUE were in the context of ammonium nitrate (Sinha et al., 2018; Subudhi et al., 2020). Therefore, in the present study, we undertook a comparative transcriptome analysis for NUE in two Indica rice genotypes with contrasting NUE, Nidhi and Panvel1, as identified earlier (Sharma et al., 2018, Sharma et al., 2021) to understand the genes/processes underlying NUE.

## Materials and Methods

### Plant Material, Growth Conditions, and Nitrate-Treatments

Two genotypes of rice (*Oryza sativa* ssp. *Indica*), namely, Nidhi and Panvel1 were chosen, based on contrasting germination, yield, and NUE (Sharma et al., 2018, Sharma et al., 2021). Seeds of Nidhi were procured from the Indian Institute of Rice Research, Hyderabad, India, whereas seeds of Panvel1 were from Panvel, Maharashtra, India. Seeds of modal weight were selected (Sharma et al., 2018) and surface sterilized using 0.1% mercuric chloride for 50 s followed by several washes with ultrapure water and allowed to soak in it for 2 h. They were sown in pots filled with nutrient-depleted sand (Sharma et al., 2019) saturated with Arnon-Hoagland medium (Hoagland and Arnon, 1950) with normal (15 mM) or low nitrate concentration (1.5 mM) as control and test conditions as described earlier (Sharma et al., 2021). The pots were replenished with media to saturation every few days and plants were grown for 21 days in the greenhouse at 28**°**C and 70% relative humidity with 270 μmol m^–2^s^–1^ light intensity and 12/12 h photoperiod. For microarray the treated and control tissues from three independent biological replicates were frozen in liquid N_2_ and stored at −80°C till further use.

### Total RNA Extraction and Microarray

The total RNA was isolated from 21-day whole plants using TRIzol reagent (Invitrogen, Carlsbad, CA, United States) as per the manufacturer’s instructions. Microarray analyses were performed under MIAME compliant conditions using independent biological triplicates. Microarray analysis was performed at Genotypic Technologies (Bengaluru, India). RNA was quantified using a NanoDrop spectrophotometer (ND2000, Thermo Fisher Scientific, Waltham, MA, United States). The integrity of the isolated RNA samples was determined by the Agilent 2100 Bioanalyzer (Agilent Technologies, Palo Alto, CA, United States) as per the manufacturer’s instructions. The ratio of 18S and 28S rRNA was obtained from 2100 Expert software (Agilent Technologies, Palo Alto, CA, United States) and the RNA integrity number was obtained from RIN Beta Version Software (Agilent Technologies, Palo Alto, CA, United States). The RNA samples used for microarray hybridization had RIN values above 6. They were reverse transcribed using 500 ng of each RNA sample into double-stranded cDNA using MMLV-RT enzyme and random primer tagged to a T7 polymerase promoter. The double-stranded cDNA was then used as a template to generate Cy3- labeled cRNA by *in vitro* transcription using RNaseOUT (Invitrogen, United States), inorganic pyrophosphatase, and T7 RNA polymerase at 40°C as per the manufacturer’s instructions [Agilent Quick Amp labeling kit (p/n:5190-0444, United States)]. Labeled cRNA was purified using Qiagen RNeasy columns (Qiagen, Cat No: Cat#74104) and assessed for yields and specific activity. Agilent Rice Gene Expression 8 × 60 K (AMADID 48014) microarrays were customized to include nuclear and organellar gene probes. Labeled cRNA samples of 600 ng each were fragmented and hybridized onto microarrays using the gene expression hybridization kit (Agilent’s *in situ* Hybridization kit 5188-5242) in an Agilent’s Surehyb hybridization chamber at 65°C for 16 h. The hybridized slides were washed and scanned using an Agilent microarray scanner.

### Microarray Data Analysis

Scanned images were processed using Agilent Feature Extraction Software (Version-11.5, United States) to obtain raw data, which were analyzed using Agilent Gene-Spring GX software (Version-12.6.1, United States). The data were normalized using the 75th percentile shift method of global normalization that adjusts the locations of all the spot intensities and provides fold expression values relative to controls. The raw and processed data were deposited in the NCBI-GEO database (GSE140257). The transcripts showing geometric mean fold change value ± 1 (log_2_FC) with statistically significant cut-of (*P* ≤ 0.05) were considered as differentially expressed genes (DEGs) in the low nitrate-treated samples relative to the normal nitrate controls. The Student’s *t*-test was used to calculate the *P*-value among the replicates. During the data analysis using R studio (Version 1.2.5042, Boston, ME, United States), we observed that one of the three biological replicates was a consistent outlier, causing either non-significant or negative correlation with the other two biological replicates and affecting the robust identification of DEGs. This problem persisted despite quantile normalization and Data-Driven Haar-Fisz for Microarrays (DDHFm) transformation and therefore, the data were re-analyzed with R studio (Version 1.2.5042, Boston, ME, United States) using raw intensity values of the best two significantly correlated replicates and used for the rest of the downstream analysis.

### Functional Classification, Subcellular Localization of Differentially Expressed Genes, and Data Analysis

Gene Ontology (GO) based functional annotation was performed using EXPath 2.0. Protein subcellular localization was predicted using the cropPAL database (Hooper et al., 2016) using default parameters for rice plants. MS Excel was used for filtering the data and the Student’s *t*-test. Venn selection^1^ was used to make Venn diagrams.

### Construction of Protein-Protein Interaction Network

The experimentally validated interactors associated with DEGs were retrieved from BioGRID,^2^ STRING,^3^ PRIN,^4^ and MCDRP^5^ databases. They were used to construct protein-protein interaction (PPI) networks in Cytoscape 3.9.0 (Shannon et al., 2003) and the expression values of DEGs were mapped onto networks. PPI subnetworks/molecular complexes were obtained using the molecular complex detection (MCODE) plugin in Cytoscape. Transcriptional regulatory networks (TRN) were developed using Cytoscape for DEG-encoded transcription factors based on rice ortholog information retrieved from Arabidopsis (Gaudinier et al., 2018). The expression values of DEGs were used to label the nodes in the networks. Expath was used for GO analysis of DEGs.

### Physiological Measurements

Potted plants grown for 21 days were used to measure photosynthesis, stomatal conductance, and transpiration rate using the LI-6400XT Portable Photosynthesis System (LI-COR Biosciences, Lincoln, NE, United States). The net photosynthetic rate was measured in terms of CO_2_ assimilated as μmol (CO_2_) m^–2^s^–1^; transpiration was measured in terms of mol (H_2_O) m^–2^s^–1^; stomatal conductance was measured in terms of mmol (H_2_O) m^–2^sec^–1^; internal water use efficiency was measured in terms of μmol CO_2_/mol (H_2_O) and transpiration efficiency was measured in terms of μmol (CO_2_)/mmol (H_2_O) m^–2^s^–1^. The Student’s *t*-test was performed on test *vs*. control data. The reference CO_2_ concentration was 410 ± 20 μmol mol^–1^ during the measurements. All LICOR measurements were carried out at the time of maximal photosynthetic activity between 12:00 p.m. and 5:00 p.m. IST. All the measurements were done in five independent replicates.

### RT-qPCR Validation of Nitrate-Responsive Expression of Differentially Expressed Genes

Total RNAs were isolated from 21 days old whole potted plants grown in normal and low nitrate concentrations (15 mM as control and 1.5 mM as a test). 3 μg each of total RNA was reverse transcribed into cDNA using PrimeScript 1st strand cDNA synthesis kit (Takara, Kusatsu, Shiga, Japan). To avoid amplification from genomic DNA, primers spanning exon-exon junctions were designed using the Quant Prime tool.^6^ The primer sequences are provided in Supplementary Table S4. Quantitative reverse transcription polymerase chain reaction (RT-qPCR) reactions were carried out in three technical replicates and two independent biological replicates in an Agilent Aria-Mx Real-Time PCR System. Each 10 μl reaction mix contained 1 μl of undiluted cDNA, 1.0 μl of forward and 1.0 μl of reverse primers (10 μM), and 5 μl of KAPA SYBR FAST Master Mix (2×) Universal (Kapa Biosystems, Wilmington, MA, United States). The relative changes in gene expression were quantified by the 2^–△△CT^ method (Livak and Schmittgen, 2001) using actin genes (*BGIOSGA013463*) as internal controls. Melting curve analyses of the amplicons were used to determine the specificity of RT-qPCR reactions. The data were statistically analyzed by unpaired *t*-test using the software MS Excel.

### Retrieval of Molecular Functions and Identification of MicroRNAs and Their Targets

Transcription factors (TFs) encoded by DEGs were retrieved from the databases PlantPAN3.^7^

For transcription factor binding sites (TFBS) prediction, 2 kb promoter upstream sequences of the translational start site of the TFs were downloaded from RAPDB and subjected to Regulatory Sequence Analysis Tools (RSAT).^8^ To find out the motif sequences 6,7 and 8 mer sequences with a significance level (*P* < 0.05) were obtained from transcription factor binding sites (TFBS). Tomtom v 5.1.1 tool^9^ (Gupta et al., 2007) with default settings was used to filter redundant motifs and define known conserved regulatory elements (CREs) based on the Arabidopsis DAP motifs database. GoMo tool^10^ was used for the identification of detection of possible biological and molecular functions (Buske et al., 2010). Transporters encoded by DEGs were retrieved from the Rice transporters database^11^ and Transport DB 2.0.^12^ Plant microRNA (miRNA) database was used to retrieve the miRNAs that target NUE-related genes (PMRD^13^). The database Plant PTM Viewer was used to finding the products of DEGs associated with post-translational modifications (PTM).^14^

### Identification of Nitrogen Use Efficiency-Genes and Their Co-localization Onto Nitrogen Use Efficiency-QTLs

Nitrogen use efficiency genes were defined as N-responsive and yield-related genes, as reported by Kumari et al. (2021). The yield-related genes reported therein were further updated from literature and databases and used for Venn selection with the N-responsive DEGs identified in the genotypes Nidhi and Panvel1 to obtain the NUE genes in them. Similarly, NUE-QTLs reported therein were further updated from literature and the NUE genes identified in Nidhi and Panvel1 were co-localized onto NUE-QTLs as described by Kumari et al. (2021).

## Results

### Nitrate-Responsive Transcriptomes of Rice Genotypes With Contrasting Nitrogen Use Efficiency

**FIGURE 1:**
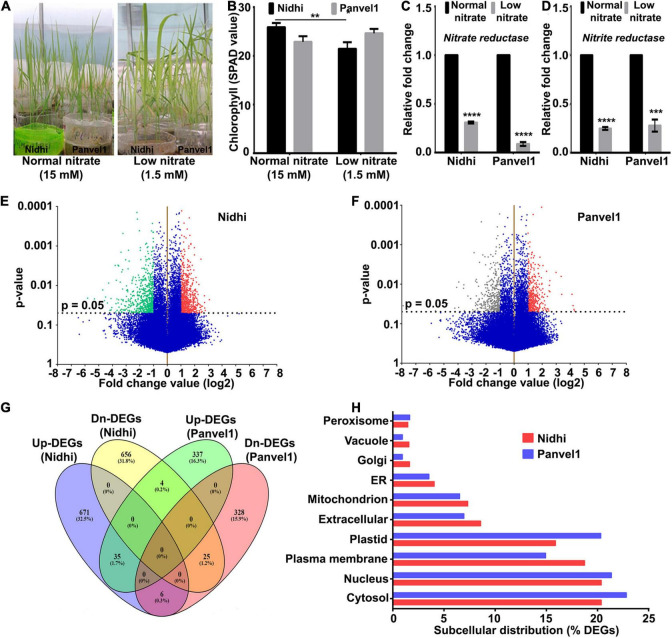
Nitrate-responsive transcriptomes in Nidhi and Panvel1. **(A)** Representative image of 21 days old Nidhi and Panvel1 plants grown in nutrient-depleted soil supplemented with AH media containing normal (15 mM) and low (1.5 mM) nitrate. **(B)** Leaf chlorophyll content (SPAD value) was estimated earlier in Nidhi and Panvel1 (Sharma et al., 2021). Quantitative RT-PCR was used to calculate the relative mRNA expression of nitrate reductase **(C)** and nitrite reductase **(D)** genes in 21 days old Nidhi and Panvel1 plants. Test samples were low nitrate, whereas normal nitrate samples were used as control. The actin gene was used as a reference gene to normalize the expression data. Data represent the mean ± SE of three technical replicates. An unpaired *t*-test was performed in GraphPad Prism. Experiments were performed repeatedly with two independent biological replicates. Volcano plots for differential gene regulation are shown for Nidhi **(E)** and Panvel1 **(F)**. Scattered dots represent different transcripts and the horizontal dashed line corresponds to the *P*-value cut-off (*P* = 0.05). Red scattered dots represent the mapping of upregulated genes, whereas downregulated genes are by green dots. **(G)** Venn diagram shows commonly and uniquely up or downregulated DEGs between Nidhi and Panvel1. **(H)** Predicted subcellular localization of DEGs encoded proteins in Nidhi and Panvel1. **P < 0.01, ***P < 0.001, ****P < 0.0001.

In this study, we used two Indica rice genotypes Nidhi and Panvel1 identified previously (Sharma et al., 2018, Sharma et al., 2021) as a contrast for their nitrate response and NUE (Figures 1A,B). They were grown in pots with low nitrate (1.5 mM for the test) and normal nitrate (15 mM for control) and total RNAs were isolated from 21 days old whole plants and used for whole transcriptome microarray analysis. Nitrate metabolism marker enzymes genes, viz., nitrate reductase (*NR*), and nitrite reductase (*NiR*) were used to assess the effects of low nitrate in both the genotypes. In both the genotypes, *NR* and *NiR* transcripts were significantly reduced in low nitrate compared with normal nitrate (Figures 1C,D). The raw microarray data were deposited in GEO at NCBI under the accession number GSE140257. After comparing the data from three independent replicates, the best two replicates with higher correlation coefficients were selected. Their scatter plots showed good correlations between the two replicates (Supplementary Figure S1). Transcripts showing geometric mean fold change value ± 1.0 (log_2_FC) with statistically significant cut-off (*p*-value ≤ 0.05) were used to identify the differentially expressed genes (DEGs). As visualized in the volcano plot (Figure 1E), 1,397 DEGs were detected in Nidhi, out of which 712 were upregulated and 685 DEGs were downregulated in response to low nitrate (Figure 1E and Supplementary Table S1). Similarly, a total of 735 DEGs were detected in Panvel1, of which 376 were upregulated while 359 were downregulated (Figure 1F and Supplementary Table S1). Many of the well-known N-regulated genes figured among the DEGs identified in this study, confirming the overall reliability of our transcriptome data (Supplementary Table S2). Interestingly, only 70 DEGs were common between these two genotypes, of which 41 were upregulated, while 29 DEGs were downregulated (Figure 1G and Supplementary Table S1). Further, Nidhi showed differential expression of many more genes than Panvel1, clearly indicating a more extensive genome-wide nitrate response in the genotype Nidhi. The sequences of proteins encoded by the DEGs were retrieved from RAP-DB and their subcellular localizations were predicted by the CropPAL2 tool (Hooper et al., 2016) using default parameters and rice as the reference organism. In the case of multiple predicted localizations, the first hit was considered. In both the cultivars, DEG-encoded proteins were predominantly located in the cytosol, followed by nucleus and plasma membranes among others (Figure 1H). Interestingly, plasma membrane-associated protein-encoding DEGs were comparatively higher in Nidhi, whereas plastid localized proteins were predominant in Panvel1 (Figure 1H).

### Nitrate Induces Common and Distinct Processes/Pathways in Contrasting Genotypes

**FIGURE 2:**
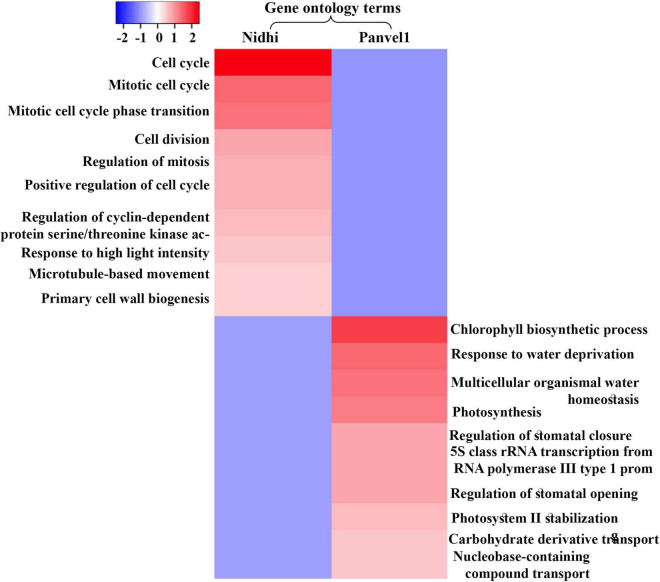
Heat map was constructed using Heatmapper, which represents enriched top ten gene ontology (GO) terms (biological processes) for contrasting rice genotypes Nidhi and Panvel1. Minus log *P* values were plotted against the respective GO term.

An important purpose of comparatively analyzing contrasting genotypes is to identify the key cellular processes involved in NUE. For this purpose, Gene Ontology (GO)-based functional annotation of DEGs was performed using EXPath 2.0 tool (Chien et al., 2015). It revealed photosynthesis, response to light stimulus, protein-chromophore linkage, and carbohydrate metabolism among others, as enriched biological processes regulated by nitrate that are common to both the genotypes (Figure 2 and Supplementary Table S3). Cell cycle/division, nitrogen utilization, ammonium transport, ammonia assimilation cycle, and processes related to cytoskeleton among others were highly enriched only in Nidhi under low nitrate conditions (Supplementary Table S3). However, biological processes related to signal transduction, protein import into the nucleus and mitochondria, phosphorelay signal transduction, chromatin organization, transport of ions, water, and carbohydrate, and response to heat and ozone among others were enriched only in Panvel1 (Supplementary Table S3).

### Validation of Selected Differentially Expressed Genes by RT-qPCR

**FIGURE 3:**
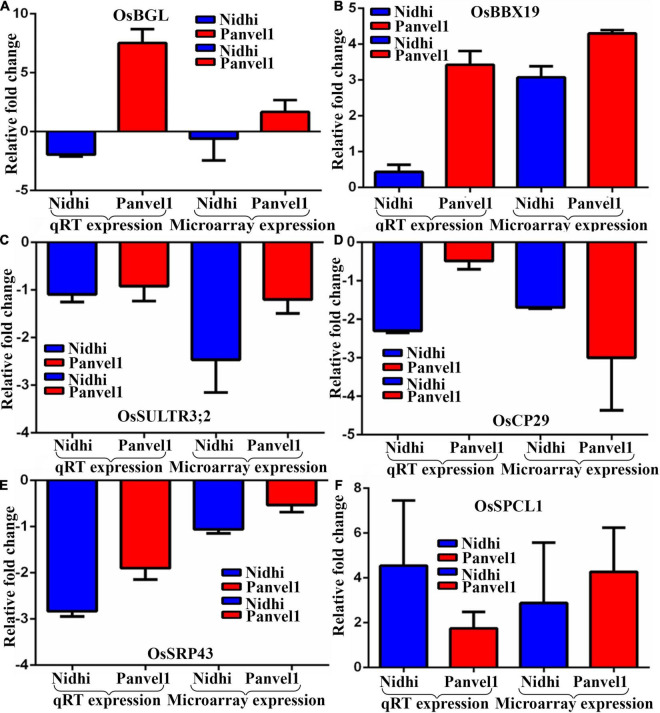
Validation of expression profile of nitrate responsive genes by RT-qPCR. Relative change in the gene expression was calculated by the comparative *Ct* value method and the actin gene was used for data normalization. The control values were taken as zero and the test values are shown as the average of three technical and two independent biological replicates (+SE) except gene *BGL1* for which the calculations were done based on three technical replicates of a biological replicate. Each sub-figure compares gene expression of RT-qPCR and microarray for Nidhi versus Panvel1 for gene *OsBGl1* **(A)**, *OSBBX19* **(B)**, *OsSULTR3;2* **(C)**, *OsCP29* **(D)**, *OsSRP43* **(E)**, and *OsPCL1* **(F)**.

The expression pattern of DEGs associated with photosynthesis, transport, and flowering time were validayted by RT-qPCR (Figure 3). One of them that is common to both the genotypes codes for B-Box-Containing Protein 19 (*OsBBX19, Os06g0298200*) and was upregulated. Two DEGs light-harvesting protein CP29 (*OsCP29, Os07g0558400*) and Chloroplast Signal Recognition Particle 43 (*SRP43, Os03g0131900*) exclusive to Nidhi were downregulated. Three DEGs were exclusive to Panvel1, two of which were upregulated: Big Grain Like 1 (*BGL1, Os03g0414900*) and Phytoclock 1 (*OsPCL1, Os01g0971800*) while sulfate transporter 3;2 (*Ossultr3;2, Os03g0161200*) was downregulated. The list of primers used in this study is provided in Supplementary Table S4.

### Differential Regulation of Transporters May Contribute to Nitrogen Use Efficiency in Contrasting Genotypes

**FIGURE 4:**
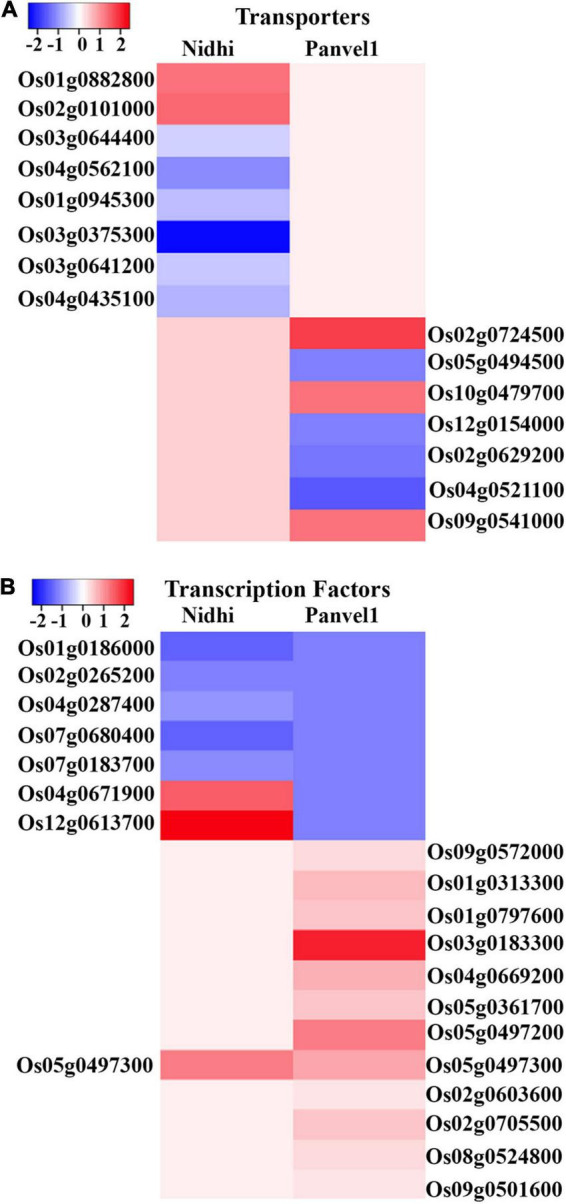
**(A)** Heat map was constructed using Heatmapper, which represents the expression pattern of nitrate regulated differentially expressed genes (DEGs) of the top two transporters’ families for Nidhi and Panvel1 rice contrasting genotypes. For Nidhi, the top two transporter families are Amino Acid/Auxin Permease (AAAP) Family and Amino Acid-Polyamine-Organocation (APC) Family, while for Panvel1 they are Drug/Metabolite Transporter (DMT) Superfamily and Major Intrinsic Protein (MIP) Family. **(B)** Expression pattern of nitrate regulated DEGs of top two transcription factors’ families for Nidhi and Panvel1 rice contrasting genotypes. For Nidhi, the top two TFs families are WRKY and ARF, while for Panvel1 they are AP2 and bHLH.

Nitrate-responsive transporters have been implicated in source-sink dynamics (Tegeder and Masclaux-Daubresse, 2018) and NUE (Wang et al., 2018; Zhang Z. et al., 2020; Kumari et al., 2021). To discriminate the effects of nitrate on different transporters in Nidhi and Panvel1, DEGs were searched in Transport DB (see text footnote 12) and Rice Transporter database and associated transporters were retrieved. Sixty-six and twenty-seven nitrate-responsive transporters belonging to 22 and 18 families, respectively, were detected in Nidhi and Panvel1 (Figure 4A and Supplementary Table S5). Two transporters belonging to distinct families, a potassium permease (*KUP; Os12g0515400*) and a sodium symporter (*DASS; Os03g0575200*), were similarly regulated in both the genotypes, whereas 64 transporters were exclusive to Nidhi and 25 were exclusive to Panvel1. Nidhi revealed a higher number of downregulated transporters than upregulated, while approximately equal numbers of up- and downregulated transporters were detected in Panvel1.

This clearly shows that nitrate differentially regulates various transporters in contrasting genotypes. The extent to which this may contribute to their differences in NUE needs to be examined, in order to consider such transporters as targets for NUE improvement. Venn analyses of these transporters found in Nidhi with NUE-related genes in rice predicted by Kumari et al. (2021) revealed nine transporters, viz. *OsCAX1a, OsTPC1, OsMGT1, OsYSL15, OsMST4, OsHAK1, OsAMT1;1, Os-YSL16 and OsMATE2* as related to NUE. Seventeen of the 66 transporters identified in Nidhi in this study were associated with biotic/abiotic stress, root, hormones, etc., whereas 40 of them are completely novel and functionally unvalidated (Supplementary Table S5). In the case of Panvel1, three transporters, viz. *OsSUT1, NPF7.1*, and *Lsi3* are associated with NUE (Kumari et al., 2021), nine were linked with other functions such as abiotic stress, micro and macronutrient transport, photosynthesis, pollen germination, etc., and 15 are completely novel and functionally unvalidated (Supplementary Table S5). Hence, our analysis identified 12 transporters as potential targets to improve NUE in rice for the first time on the basis of their differential regulation in contrasting genotypes, including 53 novel candidates (Supplementary Table S5).

### Differential Involvement of Transcription Factors and Their Binding Sites in Nitrogen Use Efficiency

To identify any differential transcriptional regulation in the contrasting genotypes for N-response/NUE, DEGs from both genotypes were searched in the rice transcription factors database PlantPAN3 (see text footnote 7). We identified 37 transcription factors (TFs) in the genotype Nidhi and 27 TFs in the genotype Panvel1, with only two TFs common to both (Figure 4B and Supplementary Table S6). In Nidhi, they belonged to 20 TF classes including 9 major classes (>2 genes) totaling 26 genes and 11 minor classes (<2 genes) totaling 11 genes. In Panvel1 the TFs belonged to 11 classes, including 5 major classes (>2 genes) totaling 21 genes and 6 minor classes (<2 genes) totaling 6 genes.

In Nidhi, all the identified members of TCP and AP2 TF families were upregulated, while the members of WRKY, bHLH, bZIP, NAC, and NAM TF families were downregulated under low nitrate. In Panvel1, AP2 and bHLH TF family members were upregulated, while only HSF TF family members were downregulated. Among the other major TF families, Myb/SANT more members were upregulated than downregulated in both the genotypes, but this was true for the ARF family only in Nidhi. In the C2H2 family, there were more members of downregulated than upregulated TFs in the genotype Nidhi, while in the genotype Panvel1, there were an equal number of up and downregulated TFs of the Homeodomain/HD-ZIP family.

Venn selection of these TFs found in Nidhi with the predicted NUE-related genes in rice (Kumari et al., 2021) revealed four TFs, viz. *OsAP2/ERF-40, OsMYB102, OsDREB1*, and *OsNAC5* as differentially regulated by nitrate, relative to Panvel1. Among them, twenty-two were associated with biotic/abiotic stress, root/leaf development, panicle architecture, hormones, etc., whereas nine of them are completely novel and functionally unvalidated (Supplementary Table S6). In Panvel1, six TFs, viz., *OsPCL1, OsBLR1, HSfA2d, R2R3-MYB, OsEPR1*, and *OsNAC3* figure among the predicted NUE-related genes (Kumari et al., 2021), while fifteen TFs were linked with other functions such as abiotic stress, spikelet meristem, stamen development, chloroplast development, and hormone metabolism, etc. Four other TFs are completely novel and functionally unvalidated (Supplementary Table S6).

These results clearly indicate that nitrate regulates common and exclusive TFs, which may control different N-response/NUE in Nidhi and Panvel1. Further, in addition to our validation of some of the predicted NUE-related TFs as differentially regulated by nitrate among contrasting rice genotypes, we identified 13 TFs as novel candidates in contrasting rice genotypes (Supplementary Table S6) to improve NUE.

Transcription factors are known to regulate target genes by binding the *cis*-acting motifs present in their promoter regions. To further discriminate the distinct N-response/NUE in the contrasting genotypes Nidhi and Panvel1, TF binding sites (TFBS) were predicted/searched using Regulatory Sequence Analysis Tools (RSAT) (see text footnote 8). Transcription factor binding sites for the transcription factors exclusively N-responsive in the genotype Nidhi revealed the majority of binding sites for AP2-EREBP and Cys2His2 (C2H2) followed by TCP, G2 like, and FAR1. This indicates that the NUE-related TFs are themselves regulated by nitrate through these families of TFs. Annotation analysis of these motifs revealed various interesting biological and molecular functions, which include regulation of transcription, translation, ATPase activity, and structural constituent of ribosome, while two motifs were not annotated. All *cis*-regulatory sites (CREs) are novel NUE-related CREs in rice (Supplementary Table S7).

In Panvel1, promoter regions for the transcription factors exclusively N-responsive in this genotype contain binding sites for GRF, AP2-EREBP followed by bzip, MYB, HSF, TCP, and Cys2His2 (C2H2). This indicates that these NUE-related TFs are themselves regulated by these families of TFs in Panvel1 as well. Annotation analysis of these motifs revealed many interesting biological and molecular functions, which include regulation of transcription, translation, response to auxin stimulus, kinase activity, and peroxidase activity, while three motifs were not annotated. All CREs identified in the present study are novel NUE-related CREs in rice (Supplementary Table S7).

**FIGURE 5:**
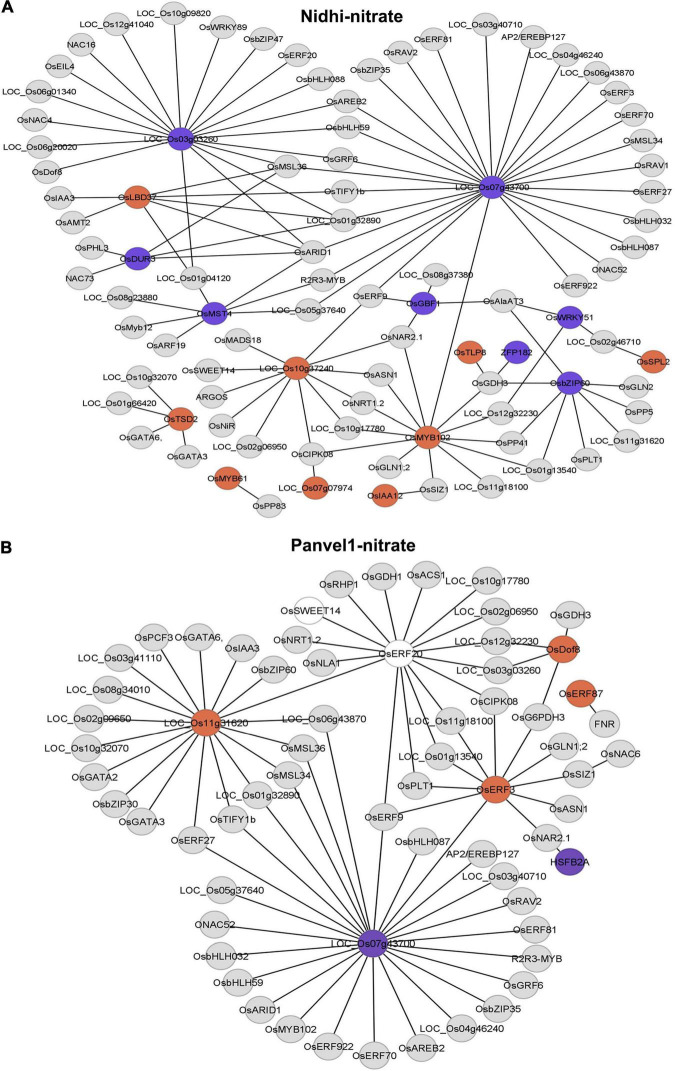
Predicated nitrate-responsive transcriptional regulatory network (TRN) in Nidhi **(A)** and Panvel1 **(B)**. Nitrate-regulated Arabidopsis TRNs (Gaudinier et al., 2018) were used to identify the DEGs-associated interactors in rice. Orthologous information was retrieved from the PlantGDB database and networks were constructed in Cytoscape ver 3.9.0. Expression values of DEGs were mapped onto the networks where red color nodes represent upregulated DEGs and blue color nodes correspond to downregulated DEGs. Light gray color nodes represent interactors but not DEGs in Nidhi and Panvel1.

To further delineate the contrasting N-response/NUE of Nidhi and Panvel1 in the context of global regulation of TFs, we predicted a DEG-associated transcriptional regulatory network (TRN). For this purpose, we used ortholog information available in Arabidopsis (Gaudinier et al., 2018) to retrieve the corresponding rice DEGs from the PlantGDB database as described earlier (Pathak et al., 2020). Then, we constructed DEG-associated TRN for Nidhi and Panvel1 in Cytoscape and mapped the expression value of DEGs onto the networks (Figure 5). Venn analysis was performed to identify the common and unique genes associated with the TRNs developed in Nidhi and Panvel1. It revealed 51 genes common to both the genotypes, whereas 42 and 15 genes were exclusive to Nidhi and Panvel1 TRNs, respectively (Supplementary Table S8). High-affinity nitrate transporter, glutamine synthetase, nodulin MtN3 family protein, and NIN protein among others were common to both the genotypes. MADS-box family gene (*OsMADS18*), auxin-regulated gene involved in organ size (*ARGOS*), and Ser/Thr protein phosphatase family protein among others were exclusive to N-response in Nidhi, whereas heat stress transcription factor B-1 and TCP family transcription factor among others were exclusively N-responsive in Panvel1 (Supplementary Table S8). Further, GO-based functional annotation of TRNs using the EXPath tool revealed response to nitrate and auxin and regulation of transcription as common GO terms in both the cultivars, whereas exclusive GO terms in Nidhi were cell differentiation, carbohydrate transport, and protein dephosphorylation, among others. Those exclusive to Panvel1 were responses to salicylic acid and cold and root development (Supplementary Table S9).

### MicroRNA-Mediated Post-transcriptional Regulation of N-Response in Contrasting Genotypes

MicroRNA are involved in the post-transcriptional regulation of gene expression in plants (Michlewski and Cáceres, 2019). To understand the possible role of post-transcriptional regulation in contrasting NUE rice genotypes, DEG-associated microRNAs were retrieved from the Plant miRNA database (see text footnote 13). A subset of nitrate-regulated genes in contrasting rice genotypes comprised 35 miRNAs targets in Nidhi and 21 in Panvel1 (Supplementary Table S10). Out of the 35 miRNA targets found in Nidhi, 22 were upregulated whereas 13 were downregulated. Similarly, out of 21 targets in Panvel1, 11 were upregulated and 10 were downregulated. Two miRNAs osa-miR167c and osa-miR441a were common between Nidhi and Panvel1. Venn analyses of these miRNAs with those targeting reported NUE-related genes in rice (Kumari et al., 2021) revealed three miRNAs osa-miR1440, osa-miR170a, and osa-miR399e exclusively in Nidhi. Overall, our analysis in contrasting genotypes identified 54 miRNAs as novel candidates (Supplementary Table S10) to be validated further for their role in improving NUE in rice.

Gene ontology analysis of target genes of miRNA in Nidhi using ExPath 2.0 revealed the role of post-translational modifications, viz., phosphorylation, de-phosphorylation, hydrolase activity, and phosphatase activity (Supplementary Table S10). Some other associated GO terms were metal ion binding, sequence-specific DNA binding, protein dimerization activity, oxidoreductase activity regulation of transcription, and DNA-template. Gene ontology for target genes of miRNA in Panvel1 showed processes such as cytosol, metal ion binding, plasma membrane, oxidation-reduction process, and protein binding. The details of their genes, functions, and gene ontology analysis along with references are provided in Supplementary Table S10. This gene ontology analysis indicates differential regulation of miRNA targets in contrasting rice genotypes and predominant post-transcriptional regulation by nitrate in the genotype Nidhi than in Panvel1.

### Genotype-Specific N-Responsive Protein-Protein Interaction Networks

**FIGURE 6:**
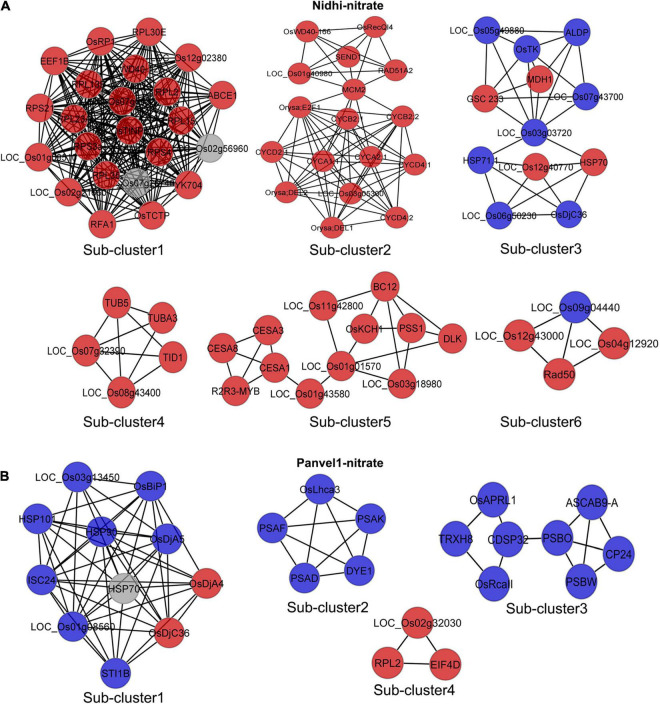
Nitrate-responsive protein-protein interaction (PPI) sub-clusters/molecular complexes in Nidhi and Panvel1. DEGs-associated interactors were retrieved by STRING, BioGRID, PRIN, and MCDRP databases. Experimentally validated interaction pairs were used to construct the PPI networks in Cytoscape (Supplementary Figures S2, S3). Molecular complexes/sub-clusters of PPI networks were identified using the MCODE plugin in Cytoscape. Thirteen and six sub-clusters/molecular complexes were detected in Nidhi and Panvel1, respectively. Important nitrate-responsive sub-clusters/molecular complexes identified in Nidhi and Panvel1 are shown **(A,B)** and the remaining are given in Supplementary Figure S4. Red and blue color nodes correspond to the up and downregulated DEGs, respectively. Light gray color nodes represent the interactors, which are not DEGs.

To understand the contrasting N-response/NUE of Nidhi and Panvel1 in terms of the underlying pathways, we developed DEG-associated PPI networks (Supplementary Figures S2, S3). Experimentally validated interactors associated with DEGs were retrieved from STING, BioGRID, MCDRP, and PRIN databases for this purpose. Nitrate-responsive PPI networks were constructed in Cytoscape and the expression value of DEGs was mapped onto the network for each genotype (Supplementary Figures S2, S3). In the case of Nidhi, the PPI network consisted of 528 nodes and 1622 edges, whereas 215 nodes and 368 edges were present in Panvel1. Venn analysis showed that 29 interactors were common in both the genotypes, whereas 500 and 186 exclusive interactors were detected in Nidhi and Panvel1, respectively (Supplementary Table S11). GO annotation of interactors involved in the PPI network revealed signal transduction, phosphorylation, cell cycle, and post-translational protein modification among others, as highly enriched exclusive GO terms in Nidhi. In Panvel1, highly enriched GO terms were a response to heat, protein refolding, water homeostasis, cell redox homeostasis, specification of floral organ identity, and protein import into mitochondrial matrix among others (Supplementary Table S12). To reduce the network complexity for better interpretation, MCODE algorithm-based sub-clustering of networks was performed in Cytoscape. We detected 13 network subclusters/molecular complexes in Nidhi, and 6 subclusters in Panvel1 (Figure 6 and Supplementary Table S4). Those with MCODE score > 3 and node number > 3 were considered for further analyses (Supplementary Table S13). In Nidhi, subcluster 1 with the highest MCODE score consisted of 23 nodes and 242 edges, whereas 11 nodes and 48 edges were present in cluster 1 in Panvel1. EXPath-based GO enrichment analyses revealed that important sub-clusters in Nidhi were primarily involved in cytoskeleton organization, cell wall, and related processes, whereas in Panvel1, they were chloroplast development and related processes (Supplementary Table S13).

### Nitrate-Regulated Differential Post-translational Modifications in Contrasting Genotypes

Initial gene ontology analysis of N-responsive DEGs in Nidhi using ExPath 2.0 revealed terms associated with post-translational modifications (PTM), viz., phosphorylation, de-phosphorylation, hydrolase activity, and phosphatase activity (Supplementary Table S14). In order to find out the N-responsive DEG-encoded proteins that can be modified post-translationally, gene ids were searched in the PTM viewer database (see text footnote 14). We found 475 IDs for Nidhi, of which a maximum number of PTMs (258) were found for phosphorylation followed by Hydroxyisobutyrylation (156), Acetylation (50), Carbonylation (20), Glycosylation (6), and one each of Malonylation, Succinylation, and Ubiquitinylation. Similarly, out of the 185 IDs for PTMs in Panvel1, the majority were Phosphorylation (85), followed by Hydroxyisobutyrylation (65), Acetylation (23), Carbonylation (9), N-glycosylation (2), and 1 for Ubiquitinylation (Supplementary Table S14). Venn analyses of these PTM genes with predicted NUE-related transporters and transcription factors in rice (Kumari et al., 2021) revealed post-translational modifications in the products of 7 DEGs of Nidhi and 3 DEGs of Panvel1. Out of these 7 genes, five were transporters and two were TFs. Out of the five transporters, four transporters, namely, *OsCAX1a, OsMGT1, OsHAK1*, and *AMT1.1* were modified post-translationally by phosphorylation, while *OsTPC1* was modified by acetylation. Out of the two TFs, *OsCOL4* was modified by acetylation while *OsAP2/ERF-40* by phosphorylation. Out of the 3 genes in Panvel1, two encoding TFs *OsPCL1* and *OsBBX26*, and one encoding transporter *OsSUT1* were modified by phosphorylation. Overall, our analysis identified 660 post-translationally modified proteins differentially regulated by nitrate in contrasting genotypes, of which the nature of PTM was known for only ten of them. The remaining 650 are novel and need detailed characterization and shortlisting for their role in NUE (Supplementary Table S14).

### Yield Association and QTL Co-localization of N-Responsive Differentially Expressed Genes Reveals Nitrogen Use Efficiency Candidates

**FIGURE 7:**
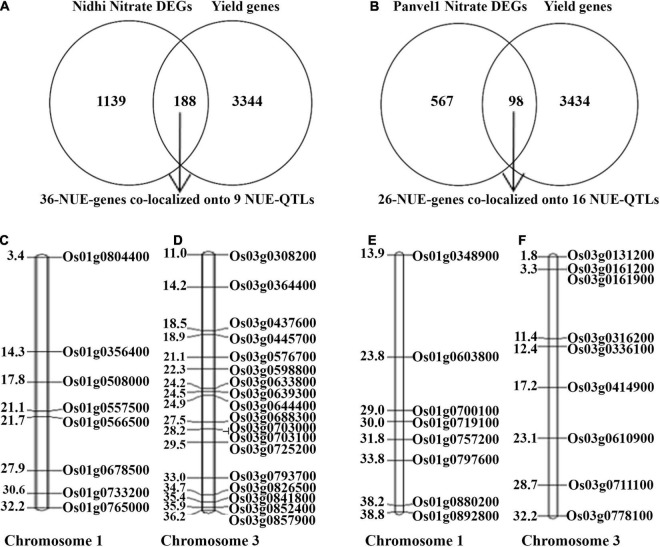
**(A)** Venn selection of yield-related and Nidhi nitrate genes revealed 188 NUE-genes. Among them, only 36 NUE-genes colocalized onto 9 NUE-QTLs. **(B)** Venn selection of yield-related and Panvel nitrate genes revealed 98 NUE-genes. Among them, only 26 NUE-genes colocalized to 16 NUE-QTLs. **(C,D)** Representative figure of Nidhi nitrate NUE genes colocalized on chromosomes 1 and 3. **(E,F)** Representative figure of Panvel nitrate NUE genes colocalized on chromosomes 1 and 3. Gene id is given on the right side of the map and the physical location of genes is given on the left side of the map (in mb).

We have earlier shown that yield association is the most important distinction between N-response and NUE, whether for the phenotype (Sharma et al., 2021) or genotype (Kumari et al., 2021). Using an updated and expanded list of 3,532 yield-related genes in rice from literature and databases and the N-responsive DEGs identified in each of the contrasting genotypes, Venn Selection was performed and the resulting common genes are termed NUE-genes. This exercise with 1,327 DEGs exclusive to Nidhi revealed 188 NUE-genes (Figure 7A). Among these, 36 NUE- related genes (Nidhi nitrate) co-localized onto 9 NUE-QTLs regulating 7 phenotypic traits including grain yield response (GR), plant height (PH), panicle length, (PL) root length (RL), relative shoot dry weight (RSW), thousand-grain weight (TGW), spikelet per primary panicle (SPY). The maximum number of 18 NUE-genes co-localized onto chromosome 3, followed by 8 on chromosomes 1, 3 on chromosome 11, and two each on chromosomes 6 and 11 (Figure 7B and Supplementary Table S15). GO analysis of these 36 NUE- genes revealed Cyano-amino acid metabolism, starch, and sucrose metabolism as important pathways at FDR ≤ 0.05 (Supplementary Table S16).

Similar Venn selection using 665 N-responsive DEGs exclusive to Panvel1 and 3532 yield-related rice genes from literature resulted in 98 NUE-genes. Among these, 26 were co-localized onto 16 NUE-QTLs regulating 11 phenotypic traits including a number of productive tillers (PTN), grain yield response (GR), harvest index (HI), nitrogen use efficiency (NUE), panicle length (PL), plant height (PH), relative biomass (RBM), relative shoot dry weight (RSW), spikelet fertility percentage (SFP), spikelet per primary panicle (SPY), and thousand-grain weight (TGW). The maximum number of 9 genes were co-localized onto chromosome 3, 8 genes on chromosome 1, 2 genes each on chromosomes 2 and 7, and one gene each co-localized onto chromosomes 4, 5, 6, 8, and 11 (Figure 7 and Supplementary Table S15). GO analysis of these 36 NUE - genes revealed photosynthesis, carbon metabolism, and pyruvate metabolism along with others as important pathways at FDR ≤ 0.05 (Supplementary Table S16).

### Nitrate Influences Photosynthetic and Water Use Efficiencies in Contrasting Genotypes

**FIGURE 8:**
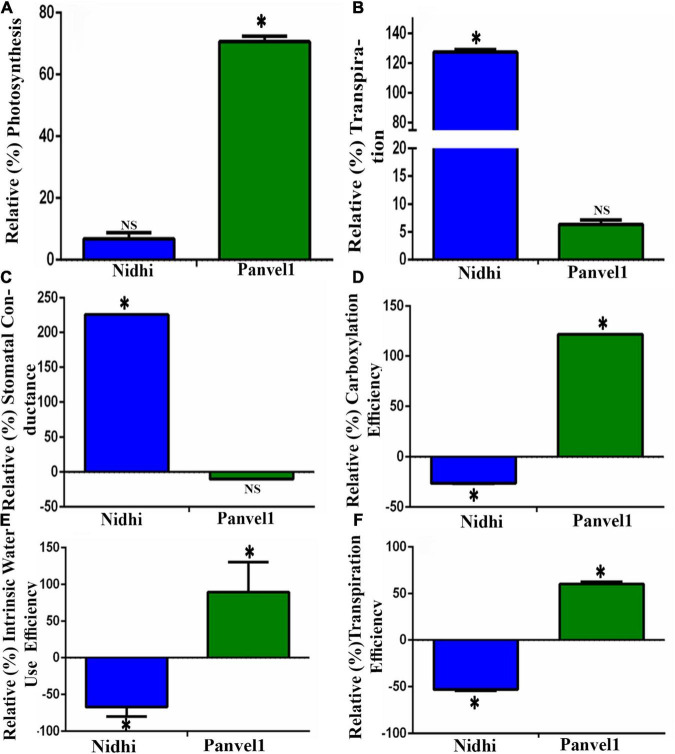
Validation of biological processes: Validation was done using Licor instrument 6400XT (LI-COR, Lincoln, NE, United States) on 21 old days grown plants. Plants were grown in nutrient-depleted soil and fertilized with Arnon Hoagland medium having nitrate as the sole source of N with 15 mM concentration as control while 1.5 mM, was used as a test. Measurement was done in five biological replicates. Percent increase or decrease (relative measurement) for each of the measurements was calculated in low nitrate over normal nitrate. **(A)** Relative photosynthesis was measured in terms of μmol (CO_2_) m^–2^s^–1^. **(B)** Relative transpiration was measured in terms of mol (H_2_O) m^–2^s^–1^, and **(C)** Relative stomatal conductance was measured in terms of mol (H_2_O) m^–2^sec^–1^). **(D)** Relative carboxylation efficiency was measured in terms of μmol (CO_2_)/m^2^s^1^/Ci as the ratio of photosynthesis and internal CO_2_ concentration, **(E)** Relative internal water use efficiency was measured in terms of μmol (CO_2_)/mol (H_2_O), and **(F)** Relative transpiration efficiency was measured in terms of μmol (CO_2_)/mmol H_2_O m^–2^s^–1^. The test of significance for low nitrate over normal nitrate for each of the individual bars has been shown as star (*P* < 0.05), while NS represents non-significance.

To experimentally validate some of the common physiological processes, viz. photosynthesis, transpiration, water stress, and stomatal conductance in the contrasting rice genotypes Nidhi and Panvel1, they were grown in the greenhouse for 21 days as described in materials and methods. They were used to measure photosynthesis, transpiration, stomatal conductance, photosynthetic efficiency, transpiration efficiency, and internal water use efficiency. For the purpose of better understanding of NUE, the relative percentage was calculated in low nitrate over normal nitrate for each of the measured parameters. All three relative efficiencies in low nitrate (1.5 mM) over normal nitrate (15 mM) were found to be significantly higher (*P* < 0.05) for the high NUE genotype Panvel1 than for low NUE genotype Nidhi (Figure 8). These results are in line with our earlier results (Kumari et al., 2021). The relative rate of transpiration and stomatal conductance were significantly higher (*P* < 0.05) in the genotype Nidhi than in Panvel1, while the opposite pattern was found in the high NUE genotype Panvel1 (Figure 8). Interestingly, relative photosynthesis was significantly higher in low nitrate in genotype Panvel1 while it was not found significant in the genotype Nidhi (Figure 8).

## Discussion

Understanding the genetic determinants of NUE is crucial for crop improvement toward NUE and much remains to be done despite the rapid recent progress in this direction (Raghuram and Sharma, 2019). This study used comparative microarray analysis of nitrate response in two Indica rice genotypes, viz. Nidhi and Panvel1 with contrasting NUE toward dissection of the genetic basis for NUE, as well as to identify its underlying biological processes. The two genotypes, namely, Nidhi and Panvel1 were previously characterized as contrasting for NUE (Sharma et al., 2018, Sharma et al., 2021). Their potted whole plants were grown in nutrient-depleted sand as described earlier (Sharma et al., 2019) to ensure precise control of the N-form (nitrate) and N-dose (1.5 or 15 mM) and avoid uncertainties typical in the field soils. The differential N-response and NUE of both the genotypes (Figures 1A,B) was confirmed by RT-qPCR analysis of the expression of nitrate reductase and nitrite reductase (Figures 1C,D). Following microarray analysis, some genes/processes that could explain the differences in NUE were identified and validated, apart from localizing them in QTLs known to be associated with NUE as discussed below.

Our previous transcriptomic analyses revealed many unreported genes/processes involved in nitrate-response in Indica rice (Pathak et al., 2020) as well as in Japonica (Mandal et al., 2022). Further, a meta-analysis of all N-responsive transcriptomes together with yield-related genes predicted and shortlisted some NUE-related genes (Kumari et al., 2021). Comparative transcriptomic analyses of contrasting genotypes are expected to take these studies to the next level, but they were confined to ammonium nitrate response so far (Sinha et al., 2018; Subudhi et al., 2020), hence the current comparative study was conducted with nitrate.

We hypothesized that contrasting NUE between genotypes can be traced to the differential expression of genes and can reveal the underlying biological processes/pathways. We found that the majority of the DEGs were unique or genotype-specific, while a small but significant fraction of the 70 common DEGs were either oppositely regulated between the genotypes, or differed in their extent of up or downregulation.

Another interesting finding was that among the 10 nitrate-responsive DEGs that were common to both the genotypes but were oppositely regulated, there was one gene that was predicted to be NUE related by Kumari et al. (2021). This gene is associated with GDP-mannose 3,5-epimerase activity (*OsGME1*, GO:0047918) and is related to both grain yield (Oryzabase) and N-response (Kumari et al., 2021). In our study, it was downregulated in the low NUE genotype Nidhi and upregulated in the high NUE genotype Panvel1, making it another attractive candidate for further validation of its role in NUE.

Photosynthesis is considered to be a key process that determines N response and NUE in crops (Long, 2020), through the regulation of associated genes in rice (Kumari et al., 2021; Sharma et al., 2021; Mandal et al., 2022). We sought to further validate these findings using contrasting genotypes in this study. Our gene ontology (GO) analyses of the nitrate-responsive biological processes (Supplementary Table S3) revealed photosynthesis/photosystem, response to light, and translation as prominently regulated by nitrate in both contrasting rice genotypes. Our physiological data validated this finding (Figure 8), with increased carbon fixation, photosynthetic efficiency, and internal water use efficiency in low nitrate relative to normal nitrate in the genotype Panvel1 compared to Nidhi. Therefore, these processes might explain, at least in part, the superior NUE of the genotype Panvel1 over Nidhi.

Similarly, our RT-qPCR data revealed differential regulation of CP29 (*LHCB4*) by nitrate in a genotype-specific manner. It has been established that along with other light-harvesting proteins, this gene is involved in energy dissipation in *Arabidopsis thaliana* (De Bianchi et al., 2008). Our results indicate the involvement of light-harvesting in NUE, in addition to carbon fixation discussed above. Interestingly, PPI networks of proteins encoded by DEGs in Nidhi and Panvel1 revealed a sub-cluster/molecular complex associated with chloroplast development/processes in Panvel1, but not in Nidhi (Supplementary Figure S3). Chloroplast development and associated processes such as N-assimilation are considered hotspots for NUE improvement in plants (Sandhu et al., 2021). Our earlier greenhouse/field experiments demonstrated that Panvel1 had higher chlorophyll contents (Sharma et al., 2021), which may in part explain its higher NUE *via* better management of N-related cellular homeostasis.

Transporters are known to regulate N-response/NUE in many crops (Mandal et al., 2018; Raghuram and Sharma, 2019; Madan et al., 2022), including rice (Pathak et al., 2020; Kumari et al., 2021; Nazish et al., 2021; Mandal et al., 2022). But they were not validated in contrasting genotypes to the best of our knowledge. In our present study, Nidhi revealed nine N-responsive transporters among many known to be associated with NUE, apart from an additional 17 linked with other functions and 40 others that are completely novel and functionally unvalidated (Kumari et al., 2021). In Panvel1, we found three nitrate-responsive transporters previously associated with NUE, nine linked with other functions, and 15 others that are completely novel and functionally unvalidated (Supplementary Table S5). Hence, our results validate the genotype-dependent expression of 55 transporters and aid in shortlisting them from many more transporters predicted to be associated with NUE by Kumari et al. (2021). Eleven of them have been independently validated for their role in NUE in rice (Katayama et al., 2009; Fang et al., 2013, 2017; Fan et al., 2014, 2016; Ranathunge et al., 2014; Chen et al., 2016, 2017; Wang et al., 2018; Gao et al., 2019; Tang et al., 2019) making the rest of our shortlisted transporters attractive candidates in future efforts to improve NUE.

Transcription factors (TFs) are known to regulate N-response/NUE in many crops and cereals (Mandal et al., 2018; Raghuram and Sharma, 2019; Pathak et al., 2020; Kumari et al., 2021; Madan et al., 2022). But they were not validated in contrasting genotypes to the best of our knowledge. In our present study, Nidhi transcriptome revealed four N-responsive transcription factors among many known to be associated with NUE, apart from an additional 22 linked with other functions and 11 others that are completely novel and functionally unvalidated (Kumari et al., 2021). In Panvel1, we found six nitrate-responsive TFs previously associated with NUE, 15 linked with other functions, and six others that are completely novel and functionally unvalidated (Supplementary Table S5). Hence, our results validate the genotype-dependent expression of 17 transcription factors and aid in shortlisting them from many more TFs predicted to be associated with NUE by Kumari et al. (2021). Seven of them have been independently validated for their role in NUE in rice (Lijavetzky et al., 2003; Kurai et al., 2011; Sun et al., 2016; Tang et al., 2019; Alfatih et al., 2020; Gao et al., 2020; Wu et al., 2021), making the rest of our shortlisted TFs attractive candidates for future efforts toward improving NUE. We also identified many enriched binding motifs for NUE-associated N-responsive TFs in the present study for the first time for further validation of their role in NUE and shortlisting the targets for crop improvement.

Transcriptional regulatory networks can be used to predict the underlying interactions in pathways that regulate various responses. They were used to construct transcription regulatory networks and study N-response/NUE in rice (Pathak et al., 2020; Mandal et al., 2022). But they were not examined in contrasting genotypes to the best of our knowledge. We used Arabidopsis orthologs information to construct nitrate-responsive TRN in both the genotypes (Figure 5 and Supplementary Table S8). The TF classes common to both the genotypes include AP2 domain-containing protein, no apical meristem protein, bZIP, and NAC domain-containing protein, and their target genes such as high-affinity nitrate transporter, glutamine synthetase, NIN protein, calcium/calmodulin-dependent protein kinases. Lactate/malate dehydrogenase was identified as a common expression of downregulated DEG in both the genotypes, whereas 16 and 7 DEGs were exclusive to TRN developed in Nidhi and Panvel1, respectively. Interestingly, the TRN captures some of the already validated individual targets in rice NUE such as nitrate reductase (Gao et al., 2019), glutamine synthetase1 (Brauer et al., 2011), and urea transporter (Beier et al., 2018), make the rest of the interactors in the TRN as attractive candidates in future efforts to improve NUE.

MicroRNAs are known to regulate N-response/NUE in a few crops (Zuluaga and Sonnante, 2019; Kumari et al., 2021). But they were not validated in contrasting genotypes to the best of our knowledge. In our present study, three N-responsive miRNAs among many known to be associated with NUE in Nidhi, apart from an additional 32 that are completely novel and functionally unvalidated (Kumari et al., 2021). In Panvel1, none of the miRNAs was found to be linked with NUE thus all 21 are completely novel and functionally unvalidated (Supplementary Table S10). Five of them have been independently reported for their role in yield (osa-miR1440; Liu et al., 2020, MIR396e and MIR396f; Zhang J. et al., 2020), phosphate starvation, and root traits (osa-miR399e; Dai et al., 2012) and NUE also including osa-miR170a (Kumari et al., 2021).

Our RT-qPCR validation of higher expression of the N-responsive DEG, big grain like1 (*BGL1*) in Panvel1 under low nitrate (relative to Nidhi), indicates its role in yield and NUE, as ectopic expression of *BGL1* leads to high yield through cell division and organ development enhancement (Lo et al., 2020). Similarly, a mutation in Phytoclock1 has been reported for early flowering in wheat (Mizuno et al., 2016) and our RT-qPCR results show lower expression of this N-responsive gene in Panvel1 relative to Nidhi. We earlier showed that flowering time is an important phenotypic trait for NUE (Sharma et al., 2021), indicating that the Phytoclock gene could also be one of the attractive candidates to manipulate NUE. Our RT-qPCR validation of the N-responsive upregulation of B-Box-Containing Protein 19 revealed far higher expression in Panvel1 relative to Nidhi. This gene has been reported for various kinds of stresses (Shalmani et al., 2019) and our results on its differential N-regulation in contrasting genotypes show the first indication of convergence between NUE and stress pathways. This is an underexplored area with considerable potential (Jangam et al., 2016).

An important caveat of transcriptome-based inferences is that they often ignore the role of post-translational modifications (PTMs). Recently, PTMs have been reported to play roles in nitrogen utilization, signal transduction, and response to sudden changes in nitrogen availability (Kumari and Raghuram, 2020). Our study revealed PTMs related to phosphorylation and ubiquitination among others, which could play their role in NUE. This is in line with the reported regulation of the ubiquitination pathway in controlling source-to-sink nitrate remobilization in Arabidopsis (Liu et al., 2017). We identified 475 N-responsive PTMs in Nidhi and 185 PTMs in Panvel1, demonstrating large differences between genotypes and opening an underexplored avenue to be tested for the role of specific PTMs in NUE.

Identification and characterization of QTL is a major driver of genetic improvement for any trait. But its progress for NUE has been slow, largely due to the poor characterization of the NUE phenotype, which became available recently for rice (Sharma et al., 2021). It was also pointed out recently that even though several QTL studies exist for NUE in rice, there is inadequate convergence between QTL and functional genomics (Kumari et al., 2021 and references cited therein). Earlier only N-responsive genes were co-localized on NUE-QTLs or yield QTLs (Chandran et al., 2016; Sinha et al., 2018) irrespective of their role in yield or NUE. But in the present study, we co-localized NUE genes (N-responsive and yield-related genes) to NUE-QTLs to identify NUE-candidates. On that basis, we found the maximum number of 18 NUE-genes in Nidhi and 9 NUE genes in Panvel1 co-localized with NUE- QTLs on chromosome 3. In addition, there were 8 NUE-genes each on chromosome 1 in both Nidhi and Panvel1. These results advance the findings of Kumari et al. (2021), who reported these two chromosomes as the hotspots for NUE-QTLs in rice. This information could be of great value to breeders. Finally, GO analysis of those NUE-related DEGs co-localized onto NUE-QTLs revealed that carbon fixation, carbon metabolism, and photosynthesis as important processes for NUE in Panvel1 relative to Nidhi, revalidating our findings based on gene expression and physiological data described in the earlier sections.

## Conclusion

Transcriptomic analysis of nitrate-response in two rice genotypes contrasting for NUE revealed differential involvement of biological processes, transporters, transcription factors and their networks, miRNAs, post-translational modifications, and NUE-candidates co-localized onto NUE QTLs in a genotype-dependent manner.

## Data Availability Statement

The datasets presented in this study can be found in online repositories. The names of the repository/repositories and accession number(s) can be found below: https://www.ncbi.nlm.nih.gov/geo/, accession number: GSE140257.

## Author Contributions

NS performed most of the experiments, analyzed the data, and wrote the first draft. SK performed NUE-gene identification and their co-localization to NUE-QTLs and TFBS ontology analysis, and helped in raising and harvesting plant tissues, RNA isolation, and RT-qPCR. DJ performed statistical and network analysis, and helped in RT-qPCR and manuscript drafting. NR helped in the planning, mentoring, and supervision of the experiments, data interpretation, edited, and finalizing of the manuscript. All authors read and approved the submitted version.

## Conflict of Interest

The authors declare that the research was conducted in the absence of any commercial or financial relationships that could be construed as a potential conflict of interest.

## Publisher’s Note

All claims expressed in this article are solely those of the authors and do not necessarily represent those of their affiliated organizations, or those of the publisher, the editors and the reviewers. Any product that may be evaluated in this article, or claim that may be made by its manufacturer, is not guaranteed or endorsed by the publisher.

## Footnotes

1
https://bioinfogp.cnb.csic.es/tools/venny/

2
https://thebiogrid.org/

3
https://string-db.org/

4
http://bis.zju.edu.cn/

5
http://www.genomeindia.org/biocuration/

6
https://quantprime.mpimp-golm.mpg.de/?page=about

7
http://plantpan.itps.ncku.edu.tw/

8
http://plants.rsat.eu

9
http://meme-suite.org/tools/tomtom

10
http://meme-suite.org/tools/gomo

11
https://ricephylogenomics.ucdavis.edu/transporter/genInfo.shtml

12
http://www.membranetransport.org/transportDB2/index.html

13 Zhang, Z., Yu, J., Li, D., Zhang, Z., Liu, F., Zhou, X., et al. (2010). PMRD: plant microRNA database. *Nucleic Acids Res.* 38, D806–D813. 10.1093/nar/gkp818

14
https://www.psb.ugent.be/webtools/ptm-viewer/experiment.php
