# Supplementary material for: Comparative Transcriptomic Analyses of Nitrate-Response in Rice Genotypes With Contrasting Nitrogen Use Efficiency Reveals Common and Genotype-Specific Processes, Molecular Targets and Nitrogen Use Efficiency-Candidates
Source: Front Plant Sci. 2022 Jun 14;13:881204. doi: 10.3389/fpls.2022.881204 (PMC9237547; doi:10.3389/fpls.2022.881204)
Supplement: Supplementary file 2 [file Image_1.PDF]

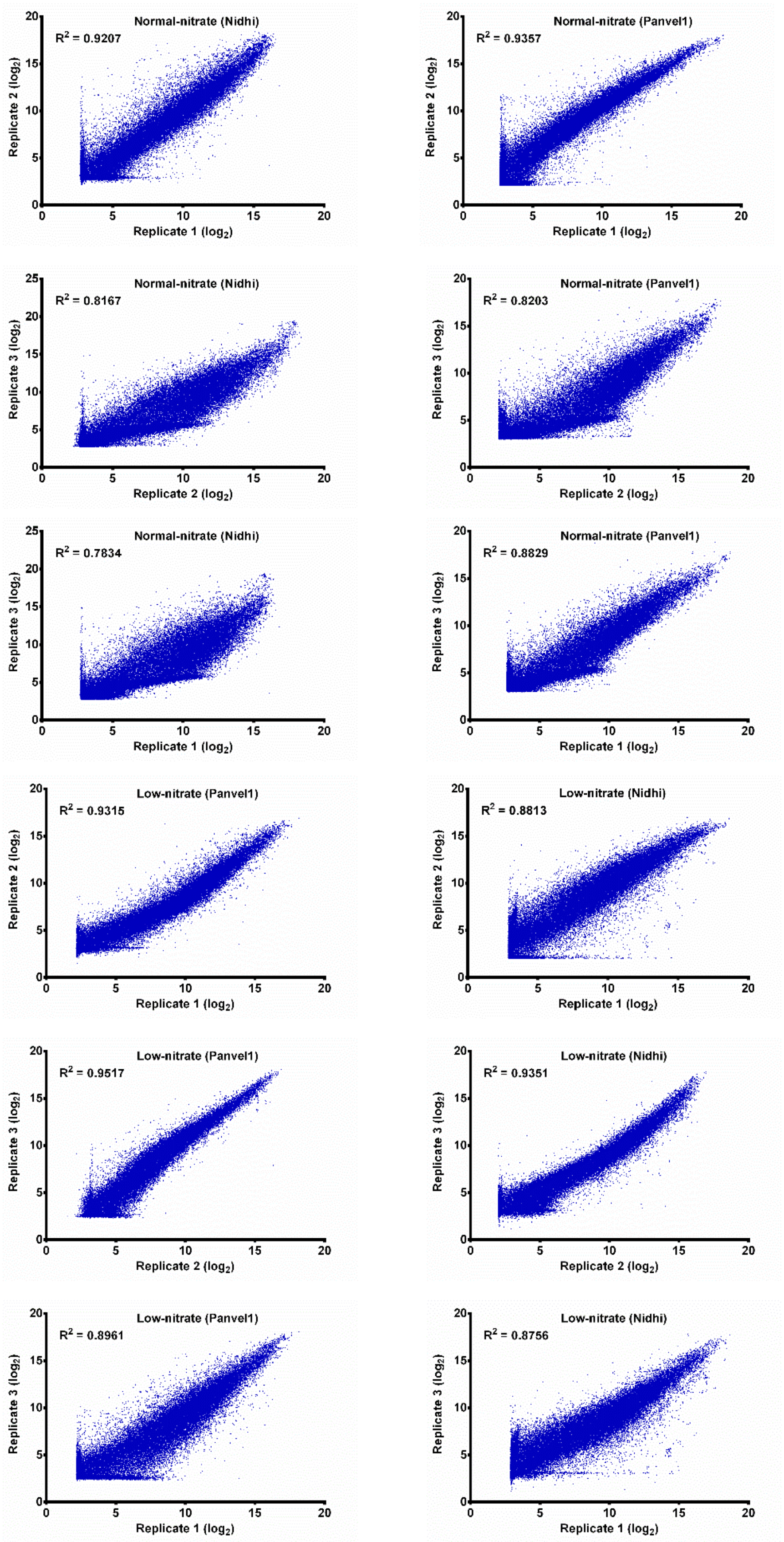

Figure S1: Scatter plots represent the correlation between the independent biological replicates. Three independent biological replicates of Nidhi and Panvel1 grown in normal- and low-nitrate were used for microarray analyses.
