# Supplementary material for: Comparative Transcriptomic Analyses of Nitrate-Response in Rice Genotypes With Contrasting Nitrogen Use Efficiency Reveals Common and Genotype-Specific Processes, Molecular Targets and Nitrogen Use Efficiency-Candidates
Source: Front Plant Sci. 2022 Jun 14;13:881204. doi: 10.3389/fpls.2022.881204 (PMC9237547; doi:10.3389/fpls.2022.881204)
Supplement: Supplementary file 3 [file Image_2.PDF]

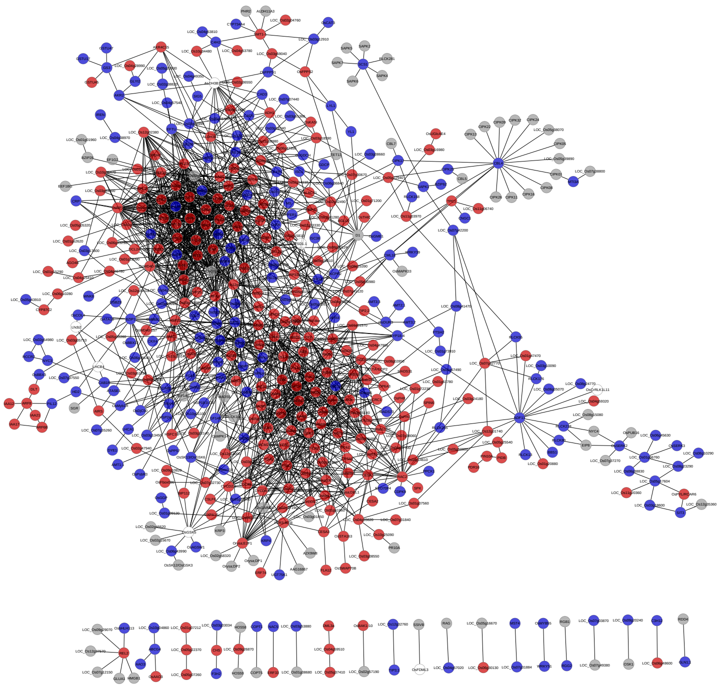

Figure S2: Nitrate-responsive protein-protein interaction networks in Nidhi. DEGs associated experimentally validated interactors were retrieved from STRING, PRIN, BioGRID and MCDRP databases. Networks were constructed in Cytoscape and expression value of DEGs were mapped onto the networks. Red nodes correspond to up-regulated DEGs whereas blue nodes represent the down-regulated DEGs. Light grey colour nodes represent interactors, which are not DEGs in this study.
