# Supplementary material for: Comparative Transcriptomic Analyses of Nitrate-Response in Rice Genotypes With Contrasting Nitrogen Use Efficiency Reveals Common and Genotype-Specific Processes, Molecular Targets and Nitrogen Use Efficiency-Candidates
Source: Front Plant Sci. 2022 Jun 14;13:881204. doi: 10.3389/fpls.2022.881204 (PMC9237547; doi:10.3389/fpls.2022.881204)
Supplement: Supplementary file 5 [file Image_4.PDF]

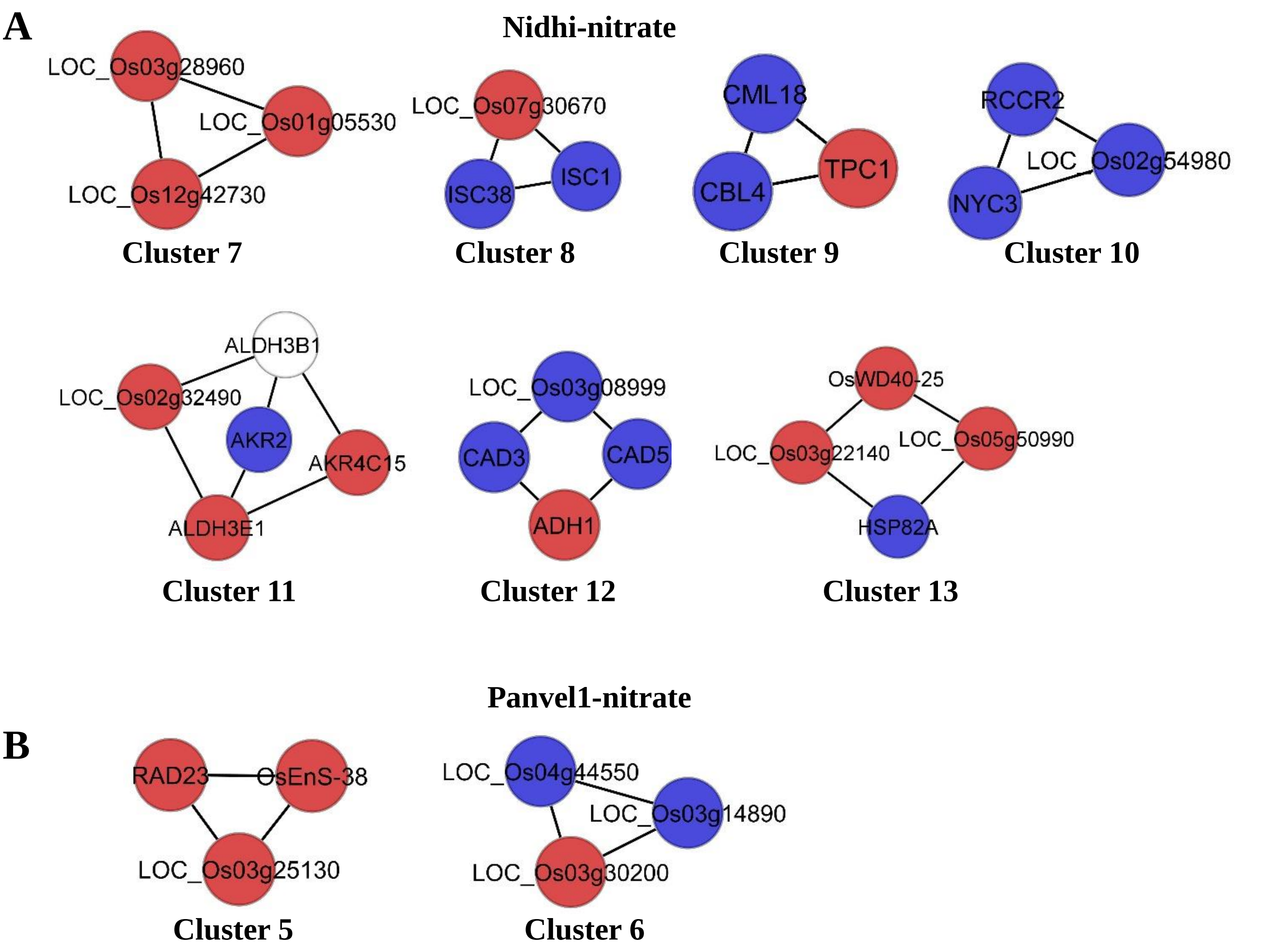

Figure S4: Protein-protein interaction (PPI) sub-clusters/molecular complexes developed in Nidhi and Panvel1. PPI networks developed in Nidhi (Figure S2) and Panvel1 (Figure S3) were subjected to sub-clustering using MCODE plugin in Cytoscape. Red nodes correspond to up-regulated DEGs whereas blue nodes represent the down-regulated DEGs. Light grey colour nodes represent interactors, which are not DEGs in this study.
